# Cross-sectional and longitudinal associations of screen time and physical activity with school performance at different types of secondary school

**DOI:** 10.1186/s12889-018-5489-3

**Published:** 2018-04-27

**Authors:** Tanja Poulain, Thomas Peschel, Mandy Vogel, Anne Jurkutat, Wieland Kiess

**Affiliations:** 1 LIFE Leipzig Research Center for Civilization Diseases, University of Leipzig, Philipp-Rosenthal-Strasse 27, 04103 Leipzig, Germany; 2 Department of Women and Child Health, Hospital for Children and Adolescents and Center for Pediatric Research (CPL), University of Leipzig, Liebigstrasse 20a, 04103 Leipzig, Germany

**Keywords:** Media consumption, Physical activity, School achievement, Type of school, Adolescents, Longitudinal

## Abstract

**Background:**

Previous studies have already reported associations of media consumption and/or physical activity with school achievement. However, longitudinal studies investigating independent effects of physical activity and media consumption on school performance are sparse. The present study fills this research gap and, furthermore, assesses relationships of the type of secondary school with media consumption and physical activity.

**Methods:**

The consumption of screen-based media (TV/video, game console, PC/internet, and mobile phone) and leisure physical activity (organized and non-organized) of 10 – to 17-year old adolescents participating in the LIFE Child study in Germany were related to their school grades in two major school subjects (Mathematics and German) and in Physical Education. In addition to a cross-sectional analysis at baseline (N = 850), a longitudinal analysis (N = 512) investigated the independent effects of these activities on the school grades achieved 12 months later. All associations were adjusted for age, gender, socio-economic status, year of data assessment, body-mass-index, and school grades at baseline. A further analysis investigated differences in the consumption of screen-based media and physical activity as a function of the type of secondary school (highest vs. lower secondary school).

**Results:**

Adolescents of lower secondary schools reported a significantly higher consumption of TV/video and game consoles than adolescents attending the highest secondary school. Independently of the type of school, a better school performance in Mathematics was predicted by a lower consumption of computers/internet, and a better performance in Physical Education was predicted by a lower consumption of TV/video and a higher frequency of non-organized physical activity. However, the association between non-organized physical activity and subsequent grades in Physical Education was significant in girls only.

**Conclusion:**

The present results suggest that media consumption has a negative effect on school achievement, whereas physical activity has a positive effect, which, however, is restricted to the subject Physical Education. Future studies might explore the relationship between media consumption and school career, for example, the choice or change of the secondary school type, in more detail.

**Trial registration:**

LIFE Child study: ClinicalTrials.gov, clinical trial number NCT02550236

## Background

Since one’s school achievement influences the following professional education and career, it has a strong impact on the future of children and adolescents. One indicator of school achievement are school grades or test scores. They have an impact on the choice of post-secondary education (e.g., different courses of studies might require different school grades) and professional opportunities. However, school achievement might also be indicated by the type of school a child attends. Different types of school differ in their level of education and in the graduation that can be achieved. The graduation, in turn, influences the further course of education (e.g., studying at a University or not) and the choice of profession. In Germany, the decision for a higher or a lower secondary education is usually made at the age of approximately 10 years. Consequently, differences in school education and career opportunities become already apparent early in a child’s development.

The school achievement of children might partly be influenced by biologically or ecologically determined aspects such as intelligence [1] or social background [2]. The way children spend their leisure time, however, might also affect their school achievement [3, 4]. Two leisure time activities that have been suggested to be associated with school achievement are media consumption, especially the consumption of screen-based media such as television and game consoles, and physical activity [5]. These activities represent popular, yet very different leisure time activities of children and adolescents.

The consumption of screen-based media is a form of sedentary behavior that has increased considerably during the last years, especially with respect to computers and mobile devices [6, 7]. Older adolescents and adolescents with a lower social background are especially prone to the consumption of electronic media [7–9]. Previous studies indicate that a high consumption of electronic media is associated with several adverse outcomes, e.g., poorer physical health and fitness [10–12], poorer mental health [13–15], and poorer school performance [5, 16–20]. Furthermore, adolescents reporting a high consumption of TV were shown to be at a higher risk of leaving school without qualification or receiving no postsecondary education than children showing a moderate consumption of TV [20, 21]. One theory that explains the association between a high media consumption and a poor school performance is the displacement hypothesis. According to this hypothesis, a high media consumption replaces other, more academic, activities, which, in turn, leads to poorer school achievement [22]. Strong support for this hypothesis results from studies showing negative relations between screen time and doing homework and social activities [23, 24]. Furthermore, recent studies could show that reduced sleeping and reading mediate the relationship between longer screen times (especially before going to bed) and poorer school achievement [22, 25]. Further factors that may mediate the relationship between a high media consumption and a poor academic performance are higher levels of sensation seeking [16], attention and learning difficulties [20], psychosocial problems [5], or negative attitudes toward school [20].

In contrast to media consumption, the physical activity of adolescents has changed only slightly over the last years [26–28]. However, most children and adolescents spend less than the recommended 60 minutes in moderate-to-vigorous physical activity per day [29–31]. Physical activity has been shown to be associated with better physical [32–34] and mental health [14, 15, 35, 36]. Furthermore, physical activity has been related to better school performance [37–44]. One focus of previous research was put on physical education classes or physical activity programs at school and their impact on academic behavior, school grades, or scores in cognitive tests. Studies in this field showed positive effects of school- or classroom-based physical activity on physical fitness, test scores, academic behavior, and school grades [37–40]. As revealed by cross-sectional [5, 41, 42] as well as longitudinal studies [43, 44], physical activity outside of the school context has also been linked to better school achievement. Factors that might mediate the positive associations between physical activity and school performance are an increase in self esteem [44], cognitive functioning and concentration [45, 46], and physical fitness [47–49].

Previous studies reported significant relations between longer screen times and lower physical activity [50, 51]. In order to better understand the interplay between both leisure activities, it is important to assess them simultaneously in the same study and to investigate independent effects of both activities. Whereas recent cross-sectional studies could show independent associations of physical activity and/or media consumption with school performance [5, 19], longitudinal studies in this field are sparse. The present study fills this research gap by assessing adolescents’ media consumption and their physical activity as potential independent predictors for subsequent school grades in German 10- to 17-year-old adolescents. In contrast to previous studies that focused on the grades in major subjects (e.g. Language, Mathematics, Science) or did not differentiate between grades in different school subjects [16, 17, 20, 41, 44], here, school grades in major school subjects (Mathematics and German) and Physical Education were distinguished. Furthermore, as school achievement is not only reflected by school grades, but also by the type of the attended secondary school, the present study also investigated associations of media use and physical activity with secondary school type. To our knowledge, this is the first study that investigates this specific relation.

We hypothesized that media consumption and physical activity show independent associations with school achievement. In more detail, adolescents attending a lower secondary school (going along with a lower educational program and fewer career opportunities) were expected to spend more time in consuming electronic media and to be physically less active than children attending a higher secondary school. In addition, a higher media consumption as well as a higher physical activity were hypothesized to show independent but inverse longitudinal associations with the school grades achieved in Mathematics, German (first language), and Physical Education.

## Methods

### Participants

The data analyzed in the present study were collected between July 2011 and April 2017 in the LIFE Child study. The LIFE Child study, a longitudinal study conducted at the Leipzig Research Center for Civilization Diseases in Leipzig, Germany, aims to examine normal child development and the development of civilization diseases [52, 53].

For the present analysis, all secondary school students who had provided information on their socio-economic status, their school grades, their attended school type, their screen time, and their physical activity at their first study visit (baseline) were considered. This sample comprised 850 10- to 17 year old adolescents (435 boys, 415 girls, mean age = 13.01).

The participating adolescents attended four different types of secondary schools, the “Gymnasium”, that is, the secondary school leading to the highest school leaving degree (in the following referred to as highest secondary school), and three other types of school (“Realschule”, “Förderschule”, and “Gesamtschule”) leading to lower secondary school degrees. As the group sizes of the latter three types of school were rather small, they were aggregated to one category (in the following referred to as lower secondary school). 68% of all participants attended the highest secondary school and 32% attended a lower secondary school.

The socio-economic status (SES) of the study sample was illustrated by an index considering the education (graduation and professional education) and occupational status of their mothers and fathers as well as the monthly family income [54]. This index ranges from 3 to 21, with higher values indicating a higher SES. Based on this index, 16% of participants belonged to the lower social milieu (index between 3 and 8.4), 62% to the middle social milieu (index between 8.5 and 15.4), and 22% to the high social milieu (index between 15.5 and 21).

For the longitudinal analysis, all participants who had participated twice – with an interval of approximately 12 months (range = 7.5 – 17.5 months) between baseline and follow-up – were considered (N = 512, 255 male, 257 female, mean age at baseline = 12.68).

### Measures

All measures were assessed via questionnaires. Participants’ screen time was assessed by asking them about the duration (hours) of the daily time spent with different screen-based media (TV/video, game console, PC/internet, mobile phone). For each medium, answers were provided on a 5-step scale (never, approximately 30 minutes, between 1 and 2 hours, between 3 and 4 hours, longer than 4 hours). For further data analysis, the response categories were transformed into durations. The following transformation algorithm was applied: never = 0, approximately 30 minutes = 0.5, between 1 and 2 hours = 1.5, between 3 and 4 hours = 3.5, longer than 4 hours = 5. A total-screen-time-score was created by summing up the durations spent with each medium.

Participants’ physical activity was assessed by asking them about the frequency of their organized physical activity (i.e., in sports clubs) and their non-organized physical activity (i.e., on an individual level). Answers were provided on a 5-step scale (never, less often than once, between 1 and 2 times, between 3 and 5 times, nearly each day). For further data analysis, these response categories were transformed as follows: never = 0, less often than once = 0.5, between 1 and 2 times = 1.5, between 3 and 5 times = 4, nearly each day = 6. The times of both, organized and non-organized physical activity, were summed up to a total-physical-activity-score.

The questionnaires on adolescents’ media consumption and physical activity were based on tools used in the KiGGS study, a large nation-wide survey on the health of German children [55–58]. Their validity could be shown in a sample of 502 participants of the LIFE Child study (50% boys, mean age = 13.5, range = 5.6-20.3) who had completed the questionnaires and had worn a BodyMedia’s SenseWear accelerometer for one week. As assessed by Spearman correlations, children who reported a longer total screen time in the questionnaire had spent more hours per day in a sedentary position (according to the accelerometer) than children who reported a shorter total screen time (*ρ* = .40, *p* < .001), while children who reported a higher physical activity in the questionnaire had spent more hours per day in moderate-to-vigorous physical activity (according to the accelerometer) than children who reported a lower physical activity (*ρ* = .29, *p* < .001).

School grades were assessed for German (= native language), Mathematics, and Physical Education. Based on the German grading system, school grades range from 1 to 6, with 1 indicating the highest, and 6 indicating the lowest school achievement. However, as only very few children reported grades 5 or 6, they were aggregated with grades 4. Consequently, grades in the present sample ranged from 1 to 4.

### Statistical analysis

To assess associations between the type of school (highest vs. lower secondary school) and screen time as well as physical activity, multiple linear regression analyses with either the total-screen-time-score or the total-physical-activity-score at baseline as dependent variable and the type of school (reference = highest secondary school) as independent variable were performed. In more detailed analyses, the regression analyses contained the single screen-based media as well as organized and non-organized physical activity as dependent variables. All associations were adjusted for the control variables age, gender, SES, year of data assessment, and body-mass-index (BMI) as well as for the other indicators of screen-based media consumption and/or physical activity. The BMI was transformed to standard deviation scores of BMI, according to the German reference norms [59].

Relations between school grades and the type of school were assessed by performing multiple linear regression analyses with grades in Mathematics, German, or Physical Education as dependent variables and type of school as independent variable. Each association was adjusted for the control variables as well as the other school grades.

Associations of screen time and physical activity with grades in Mathematics, German, and Physical Education were investigated by multiple linear regressions including the total-screen-time-score and the total-physical-activity-score at baseline as independent variables and the school grades at baseline (cross-sectional analysis) or follow-up (longitudinal analysis) as dependent variables. For the longitudinal assessment, more detailed analyses included the single screen-based media and organized and non-organized physical activity as independent variables. All associations were adjusted for the control variables and the attended type of school. In the longitudinal analyses, the associations were additionally adjusted for school grades at baseline.

All statistical models were checked for interactions between the independent variables and the control variables age, gender, and SES. An interaction was included in the model if both of the following two preconditions were met. First, the interaction had to be significant (*p* < .05). Second, the model quality had to be preserved, i.e., the interaction term did not cause a severe inflation of variance (variance inflation factor < 5). If these conditions were not met (*p* > .05 or variance inflation factor > 5), the interaction was not included in the model and, consequently, not reported.

## Results

### Descriptive analysis

Table 1 summarizes the average consumption times of the different screen-based media, the physical activity, and the school achievement of the participating adolescents. Information is provided for adolescents attending the highest vs. lower secondary schools separately. As can be seen, adolescents spent approximately 5 hours per day in the consumption of different screen-based media (*M* = 5.31, *SD* = 3.66). The average consumption time of a single medium ranged from 0.71 (*SD* = 1.14) for game consoles to 1.66 (*SD* = 1.29) for TV/video. Furthermore, adolescents reported 1.76 (*SD* = 1.73) weekly training sessions for organized physical activity and 2.17 (*SD* = 2.02) weekly training sessions for non-organized physical activity. All in all, they were physically active approximately 4 times per week (*M* = 3.93, *SD* = 2.83). With regard to school achievement, most of the adolescents reported “good” (grade 2) or “satisfactory” (grade 3) performances in Mathematics (*M* = 2.64, *SD* = 0.85), German (*M* = 2.34, *SD* = 0.76), and Physical Education (*M* = 2.16, *SD* = 0.86). Depending on the school subject, 7% to 23% of the participants reported “very good” (grade 1) performances, and only 6% to 17% of the participants reported “sufficient” (grade 4) or worse performances.

**Table 1:** Description of the assessed variables at baseline by type of secondary school

	Total	Highest secondary school	Lower secondary schools	Possible range
	(n = 850)	(n = 574)	(n = 276)	
	*M (SD)*	*M (SD)*	*M (SD)*	
Consumption of screen-based media (hours/day)				
TV/video	1.66 (1.29)	1.27 (1.13)	2.08 (1.49)	0-5
Game console	0.71 (1.14)	0.54 (.91)	1.05 (1.46)	0-5
PC/internet	1.50 (1.41)	1.44 (1.34)	1.64 (1.54)	0-5
Mobile phone	1.42 (1.65)	1.33 (1.60)	1.61 (1.75)	0-5
Total screen time	5.31 (3.66)	4.79 (3.29)	6.38 (4.15)	0-20
Physical activity (training sessions/week)				
PA organized	1.76 (1.73)	1.91 (1.74)	1.43 (1.67)	0-6
PA non-organized	2.17 (2.02)	2.18 (1.99)	2.15 (2.08)	0-6
Total physical activity	3.93 (2.83)	4.10 (2.81)	3.58 (2.88)	0-12
School grades				
Mathematics	2.64 (0.85)	2.53 (0.85)	2.89 (0.80)	1-4
German	2.34 (0.76)	2.20 (0.74)	2.61 (0.72)	1-4
Physical Education	2.16 (0.86)	1.98 (0.78)	2.53 (0.90)	1-4

Better school achievement is represented by lower school grades (following the German grading system). *PA* physical activity

### Associations of type of secondary school with screen time and physical activity

As revealed by the linear regression analysis, the total-screen-time-score differed significantly as a function of the attended type of secondary school (*b =* 1.11, *p* < 0.001). Adolescents attending a lower secondary school reported a significantly longer total screen time (*M* = 6.38, *SD* = 4.15) than adolescents attending the highest secondary school (*M* = 4.79, *SD* = 3.29). The more detailed analyses revealed that especially the consumption of TV/video and game consoles differed as a function of the secondary school type (see Tables 1 and 2). The consumption of PC/internet and mobile phones, in contrast, did not differ significantly between the highest and the lower secondary school types (see Tables 1 and 2).

**Table 2:** Associations of the consumption of different screen-based media and physical activities with type of school (highest vs. lower secondary school) at baseline

		Dependent variables: Screen-based media and physical activity					
Independent variable		TV/video	Game console	PC/internet	Mobile phone	PA organized	PA non-organized
Type of school ^a^	*b* *CI (95%)*	0.25** 0.06 - 0.44	0.27** 0.10 - 0.43	-0.08 -0.28 - 0.12	0.17 -0.06 - 0.41	-0.25 -0.53 - 0.03	0.20 -0.13 - 0.52

All associations are adjusted for gender, age, SES, year of data assessment, BMI, and other indicators of screen-based media consumption and physical activity.

*b* = regression coefficient, non-standardized, *PA* physical activity

^a^reference = highest secondary school

**p < 0.01

The analyses furthermore showed that the type of school was not significantly related to the total-physical-activity-score (*b* = -0.09, *p* = 0.69). As revealed by the more detailed analyses, neither organized nor non-organized physical activity differed significantly between the highest and the lower types of secondary school (see Tables 1 and 2).

The analysis of the relationships between type of secondary school and the respective school grades showed that the grades achieved for the subjects German and Physical Education at the highest secondary school were significantly better than the grades achieved at lower secondary schools (*b* = 0.14, *p* < 0.01 for German, and *b* = 0.18, *p* < 0.01 for Physical Education). For grades in Mathematics, the same trend was observed but did not reach significance (*b* = 0.10, *p* = 0.10).

### Associations of screen time and physical activity with school grades

As revealed by the cross-sectional analysis on associations of screen time and physical activity with school grades at baseline, a better school achievement in the major school subjects was significantly associated with lower total-screen-time-scores (*b* = 0.02, *p* < 0.05 for Mathematics and *b* = 0.02, *p* < 0.01 for German). The total-physical-activity-score, in contrast, showed no significant associations with school achievement in Mathematics (*b* = -0.01, *p* = 0.75) and German (*b* = 0.01, *p* = 0.27). Better school grades in Physical Education were significantly related to higher total-physical-activity-scores (*b* = -0.08, *p* < 0.001). The total-screen-time-score, on the other hand, showed no significant association with the achievement in Physical Education (*b* = 0.01, *p* = 0.23).

**Fig. 1:**
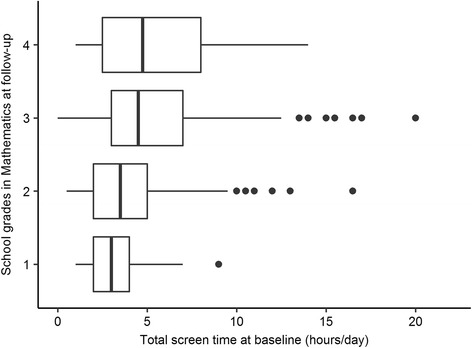
Association of total screen time at baseline with grades in Mathematics at follow-up.

The longitudinal data analysis showed that better school grades in Mathematics at follow-up were predicted by lower total-screen-time-scores at baseline (*b* = 0.03, *p* > 0.01, see also Fig. 1). As revealed by a more detailed analysis (see Table 3), the consumption of PC/internet was the major cause for this association (*b* = 0.07, *p* < 0.05). Each additional hour spent in front of the computer was associated with a lowering of the Mathematics grade by 0.07 points. Adolescents’ physical activity at baseline, on the other hand, did not predict subsequent school grades in Mathematics. Neither the total-physical-activity-score (*b* = 0.01, *p* = 0.32) nor the frequency of organized or non-organized physical activity (see Table 3) showed a significant association with school grades in Mathematics.

**Table 3:** Longitudinal analysis: Prediction of school grades at follow-up by the consumption of different screen-based media and different forms of physical activity at baseline

	Outcomes at follow-up: school grades in					
Predictors at baseline	Mathematics		German		Physical Education	
	*b*	*CI (95%)*	*b*	*CI (95%)*	*b*	*CI (95%)*
TV/video	-0.01	-0.06 - 0.05	-0.02	-0.07 - 0.03	0.07**	0.02 - 0.13
Game console	0.04	-0.02 - 0.11	0.02	-0.04 - 0.08	0.01	-0.06 - 0.07
PC/internet	0.07*	0.01 - 0.12	0.04	-0.01 - 0.09	0.02	-0.03 - 0.07
Mobile phone	0.01	-0.03 - 0.06	0.01	-0.04 - 0.04	-0.02	-0.06 - 0.02
PA organized	-0.01	-0.04 - 0.03	-0.02	-0.05 - 0.01	-0.03	-0.06 - 0.01
PA non-organized	0.03	-0.01 - 0.05	-0.01	-0.03 - 0.02	-0.07***	-0.11 - -0.04^a^

Better school achievement is reflected by lower school grades (following the German grading system). All associations are adjusted for gender, age, SES, year of data assessment, BMI, type of school, the other predictors, and grades at baseline.

*b* = regression coefficient, non-standardized, *PA* physical activity

^a^in girls only, as revealed by a significant interaction between non-organized PA and grades in Physical Education

* *p* < 0.05, ** *p* < 0.01, *** *p* < .001.

Achievement in German could be predicted neither by the total-screen-time-score (*b* = 0.01, *p* = 0.19) nor by the total-physical-activity-score at baseline (*b* = -0.01, *p* = 0.21). The more detailed analysis revealed that none of the single screen-based media or physical activities showed a significant association with school grades achieved in German (see Table 3).

**Fig. 2:**
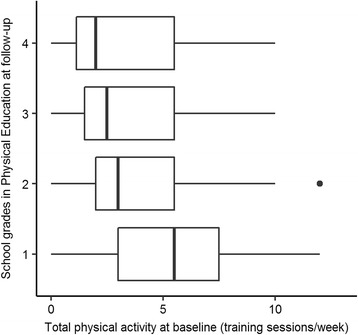
Association of total physical activity at baseline with grades in Physical Education at follow-up.

**Fig. 3:**
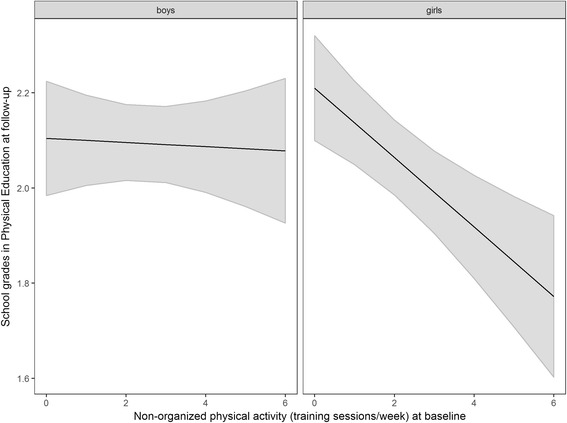
Association of non-organized physical activity at baseline with grades in Physical Education at follow-up as a function of participants’ gender.

Better performance in Physical Education at follow-up was predicted by higher total-physical-activity-scores at baseline (*b* = -0.04, *p* < 0.01, see also Fig. 2). The detailed analysis revealed a significant interaction between non-organized physical activity and gender (*b* = -0.07, *p* < .05). This interaction indicates that the baseline frequency of non-organized physical activity was associated with a better performance in Physical Education at follow-up in girls (*b* = -0.07, *p* < .001), but not in boys (*b* = -0.01, *p* = 0.82, see also Fig. 3). In contrast to non-organized physical activity, organized physical activity showed no association with grades in Physical Education at follow-up (*b* = -0.03, *p* = 0.12). Interestingly, better achievement in Physical Education was also predicted by lower total-screen-time-scores (*b* = 0.02, *p* < 0.05). The more detailed analysis (Table 3) showed that a higher consumption of TV/video was significantly associated with a lower school performance in Physical Education one year later (*b* = 0.07, *p* < 0.01).

In summary, these results demonstrate that a better school achievement in Mathematics at follow-up was predicted by shorter screen times, especially a shorter duration of PC/internet usage, at baseline. Better achievement in Physical Education was predicted by higher physical activity and shorter screen-times, especially a shorter TV consumption, at baseline.

## Discussion

The present study investigated the relationship of two frequently pursued but strongly different leisure time activities, namely the consumption of screen-based media and physical activity, with the school achievement of adolescents. It is important to note that the quality of the school achievement was represented by school grades but also by the type of secondary school attended by the adolescents.

### Associations of screen time and physical activity with school grades

Regarding the associations between leisure time activities and school achievement as indicated by school grades, we expected independent cross-sectional as well as longitudinal associations of media consumption and physical activity with school grades in Mathematics, German, and Physical Education. These hypotheses could largely be confirmed. Overall, the findings suggest a negative effect of extended screen times and a positive effect of physical activity, which, however, seems to be restricted to achievement in Physical Education.

In line with the hypothesis and previous study results [5, 16–20], children reporting a longer screen time showed lower school achievement in major subjects, that is, in Mathematics and German. The longitudinal analysis added to this finding by demonstrating negative associations between a long screen time and subsequent school achievement in Mathematics, independently of physical activity and school grades at baseline. As proposed by the displacement hypothesis, a possible reason for this finding is that adolescents’ screen time limits the amount of time spent in more academic activities such as learning or doing homework [22–24]. Another reason might be that a prolonged screen time causes learning and attention deficits or negative attitudes toward school that, in turn, lead to poorer school achievement [20].

Interestingly, the analysis of independent effects of the different screen-based media revealed that the consumption of computers/internet, but not of other media, showed a negative association with later school achievement in Mathematics. Given that a computer may be used for homework, homework related internet research, etc., this result is surprising. However, the present study did not assess the purpose of the respective computer and/or internet usage. Instead of using computers for academic purposes, adolescents might be distracted by the wide-ranging and potentially negative content provided by the internet. This assumption is supported by a study showing a negative association between excessive internet surfing and school performance [5]. Furthermore, whereas the other media might be shared with others, using computers is rarely accompanied by direct interaction with others. This lack of social interaction might have a negative impact on friendship and social integration at school, which, in turn, might have negative consequences for school achievement.

In addition to the negative association of screen time with later school achievement in a major school subject (Mathematics), screen time was also negatively related to later school grades in Physical Education. The more detailed analysis revealed that especially the time spent in front of the television was associated with lower school achievement in Physical Education, independently of adolescents’ BMI and leisure physical activity. During television viewing, the body relaxes and is nearly inactive. Long phases of inactivity might reduce the general physical fitness and muscle strength [60] which, in turn, might lead to declined performances in Physical Education at school.

With respect to physical activity, the present study showed, as expected, significant associations between the amount of time adolescents spent in physical activity and school grades in Physical Education. The longitudinal analysis furthermore demonstrated a positive association of physical activity with later school achievement in Physical Education, independently of screen time and school grades at baseline. This finding suggests that leisure time sports may improve adolescents’ general physical fitness [60], and, therefore, their performance in Physical Education at school [47–49]. An analysis of independent associations of organized and non-organized physical activity revealed that only non-organized physical activity showed an association with later grades in Physical Education, and that this association was only observable in girls, not in boys. A possible reason for this finding are differences in the form of sport girls and boys are performing in their free time. The present study did not assess the kind of sport performed. However, gender differences have already been reported in other studies [61, 62]. Sports performed more frequently by girls (e.g., dancing, aerobics, gymnastics) might better match the content of Physical Education classes or better promote the corresponding abilities than sports typically performed by boys (e.g., weight lifting, football).

Contrary to the hypothesis and other studies reporting positive associations between physical activity and general school achievement, i.e., in major school subjects [5, 41–44], here, physical activity was not associated with subsequent school achievement in Mathematics and German. These findings may suggest that the media-independent influence of leisure physical activity is restricted to achievement in Physical Education, and that the major factor guiding effects of physical activity on school achievement is an improvement of fitness, but not necessarily an improvement of attention or concentration.

### Associations of type of secondary school with screen time and physical activity

Regarding the relationship between leisure time and type of school, the present study showed that adolescents attending the highest secondary school report shorter screen times, especially with respect to television viewing and the consumption of game consoles, than adolescents attending a lower secondary school, independently of their social background and physical activity. In contrast, the frequency of physical activity did not differ between higher and lower secondary schools. These findings might be explained by differences in the daily routines of children attending different secondary schools. Given the higher learning effort at the highest compared to lower secondary schools, children attending the highest secondary school have less free time, which might manifest especially in a reduced consumption of entertaining media (TV and game console) rather than in a reduced physical activity. Another possible explanation is that a higher consumption of media increases the risk to attend a lower secondary school rather than the highest secondary school. Future studies might investigate the consumption of screen-based media at preschool level and its possible impact not only on grades received in preschool, but also on the choice of secondary school type and possible changes (from higher to lower or vice versa) between these different types of school.

### Strengths and limitations

The longitudinal character, the simultaneous exploration of screen time and physical activity, and the investigation of school achievement as indicated by school grades and type of school represent strengths of the present study. However, some limitations have to be mentioned, especially with respect to the measurement methods. All data were based on self-reports, and the given answer categories were rather broad. For collecting the media consumption data, neither the content nor the purpose of individual media consumption was assessed. For example, whether media are used for homework vs. pleasure might have a significant impact on the associations between media usage and school achievement. For physical activity, no information on the duration and intensity of doing sports was collected. Future studies may provide a more in-depth analysis of adolescents’ leisure time and apply subjective as well as objective measures, e.g., accelerometers. Furthermore, whereas the present study focused on the consumption of screen-based media only, prospective investigations might include the consumption of books and journals whose impact may differ substantially from the impact of screen-based media.

## Conclusions

The present study revealed longer screen times for adolescents attending lower secondary schools as compared to adolescents attending the highest secondary school and showed a negative association of screen time with school achievement one year later, independently of adolescents’ physical activity, age, gender, and socio-economic status. These results suggest that the consumption of screen-based media contributes to adolescents’ success at school and underline the importance to teach adolescents a reasonable consumption of screen-based media. Physical activity was associated with subsequent school achievement in Physical Education only, suggesting that the impact of physical activity may be limited to school subjects that are related to physical fitness.

## Abbreviations

BMI: Body-mass-index
PA: Physical activity
SES: Socio-economic status
